# Prostratin exhibits both replication enhancing and inhibiting effects on FIV infection of feline CD4^+^ T-cells

**DOI:** 10.1016/j.virusres.2012.11.004

**Published:** 2013-01

**Authors:** Chi Ngai Chan, Elizabeth L. McMonagle, Margaret J. Hosie, Brian J. Willett

**Affiliations:** MRC-University of Glasgow Centre for Virus Research, Glasgow, United Kingdom

**Keywords:** FIV, feline immunodeficiency virus, HIV-1, human immunodeficiency virus-1, PKC, protein kinase C, IL-2, interleukin-2, PMA, phorbol myristate acetate, FIV, Protein kinase C, Prostratin, IL-2, HIV-1, CD4^+^ T-cells

## Abstract

The phorbol ester Prostratin may either stimulate or inhibit human immunodeficiency virus-1 (HIV-1) replication. Here we report that Prostratin also exhibits a similar dual action upon feline immunodeficiency virus (FIV) replication in an IL-2-dependent feline CD4^+^ T-cell line (MYA-1). While withdrawal of IL-2 halted FIV spread, Prostratin rescued virus production and cell viability, mimicking the functions of the cytokine. Conversely, FIV grew rapidly in the presence of IL-2 and this was inhibited by Prostratin. In contrast to HIV-1, Prostratin mediated inhibition of FIV through means other than blocking virus entry. Co-application of the protein kinase C (PKC) inhibitor Gö6850 with Prostratin reversed both the inhibitory and stimulatory effects, suggesting that PKC is crucial for FIV replication.

## Highlights

► Prostratin reactivates productive infection from FIV infected, IL-2-depleted feline CD4^+^ T-cells. ► When the infected cells were supplemented with IL-2, Prostratin inhibits productive infection with FIV. ► Dual action of Prostratin on FIV-infected cells mirrors the effect of the compound on human cells infected with HIV-1. ► Dual action of Prostratin on FIV is dependent on PKC. ► Prostratin's inhibitory effect on FIV is not due to a block at viral entry, contrary to results from HIV-1 studies.

## 1 Introduction

Almost three decades after the discovery of the human immunodeficiency virus (HIV), acquired immunodeficiency syndrome (AIDS) remains an incurable disease and a major public health concern globally (Wainberg and Jeang, 2008). While anti-retroviral therapies may curtail viral replication within the infected host, considerable research effort is now being directed towards finding a way to clear the latent viral reservoirs within the infected individual in order to effect a cure for the disease (Lewin et al., 2011). Prostratin is a non-tumour-promoting phorbol ester that stimulates protein kinase C (PKC) and exhibits both stimulatory and inhibitory effects on HIV-1 replication (Biancotto et al., 2004; Brooks et al., 2003; Gulakowski et al., 1997; Gustafson et al., 1992; Korin et al., 2002; Kulkosky et al., 2001; Rullas et al., 2004; Warrilow et al., 2006). Treatment with compounds such as Prostratin is thus being targeted as a means by which latent reservoirs may be activated within the host, enabling them to be targeted for elimination. There is a pressing need for model systems in which prospective compounds for use in the elimination of viral reservoirs may be evaluated. In this study we investigate the effect of Prostratin on the *in vitro* replication of the feline immunodeficiency virus (FIV), a lentivirus that is closely related to HIV-1 and which causes an AIDS-like disease in cats (Pedersen et al., 1987).

## 2 Methods

### 2.1 Cells, virus and reagents

Feline CD4^+^ T-cells (MYA-1 cells) (Miyazawa et al., 1989) were cultured in RPMI 1640 medium supplemented with 10% foetal bovine serum (FBS), 2 mM glutamine, 100 IU/ml penicillin, 100 μg/ml streptomycin and 50 μM 2-mercaptoethanol. Actively growing MYA-1 T-cells were also supplemented with conditioned medium from a murine cell line (L2.3) transfected with a human IL-2 expression construct (equivalent to 100 U/ml of recombinant human IL-2). All media and supplements were obtained from Invitrogen Life Technologies Ltd. (Paisley, United Kingdom) except for the FBS, which was supplied by Perbio (Northumberland, UK). FIV strain GL8 (Hosie and Jarrett, 1990) was propagated in MYA-1 T-cells and stored at −80 °C prior to use. Phorbol myristate acetate (PMA) was supplied by Merck Chemicals UK (Nottingham, UK) and Prostratin by Sigma–Aldrich (Gillingham, UK). The PKC inhibitor Gö6850 was supplied by Tocris (Bristol, UK). Raltegravir (RGV) was obtained from Selleck Chemicals (Houston, TX, USA). Zidovudine (AZT) was supplied by Sigma–Aldrich.

### 2.2 Nucleic acid extraction

The Qiagen (Crawley, UK) DNA Mini kit was used to extract total cellular DNA in assays. Supernatant FIV RNA was purified from 1 ml of culture supernatant using the Qiagen UltraSens Virus kit. An internal control in the form of purified cellular RNA from un-infected feline CD4^+^ T-cells was added to all supernatant samples during the extraction process. This was to provide a way to measure the efficiency of extraction in order to adjust the estimated FIV RNA copy number per ml of supernatant. Extracted RNA was further processed by the Qiagen RNeasy Mini kit to remove contaminating FIV DNA. Manufacturer's protocols were followed. Contamination of the extracted RNA were not detected by qPCR in most sample at all the time points, the exceptions being samples from the IL-2 supplemented, productively infected cells at day 7 and day 10 post infection. The quantities of the contaminated DNA were less than 0.01% of the quantity of FIV cDNA detected.

### 2.3 Quantitative polymerase chain reaction (qPCR)

Intracellular FIV DNA and supernatant FIV RNA from infected MYA-1 T-cells were quantified using qPCR. Purified RNA was reverse transcribed into cDNA using the Roche (Welwyn Garden City, UK) Transcriptor High Fidelity cDNA synthesis kit. Manufacturer's protocol was followed and RNA was reverse transcribed using the random hexamer primer. The PCR primers FIV1360F (5′-GCAGAAGCAAGATTTGCACCA-3′) and FIV1437R (5′-TATGGCGGC CAATTTTCCT3′) plus the Taqman probe FIV1416P (5′-FAM-TGCCTCAAG ATACCATGCTCTACACTGCA-TAMRA-3′) were used to amplify a 78 bp section of the FIV *gag* gene. Primers and probes which amplify feline 18S rRNA (343-fwd: 5′-CCATTCGAACGTCTGCCCTA-3′; 409-rev: 5′-TCACCC GTGGTCACCATG-3′, and probe: 5′-FAM-CGATGGTAGTCGCCGTGCCTA-TAMRA-3′) were used as internal control. The primers, probes and templates were combined with TaqMan Universal Master Mix (Applied Biosystems, Paisley, UK) to a final volume of 20 μl per reaction in MicroAmp Optical 96-well reaction plates (Applied Biosystems). Thermo cycling was performed with an initial denaturing step at 95 °C for 5 min, followed by 40 cycles of denaturation at 95 °C 15 s; annealing and detection at 55 °C for 60 s and measurements were taken using an ABI 7500 thermal cycler (Applied Biosystems). Results were analysed using the Sequence Detection Software v1.4 (Applied Biosystems). Relative quantities of expression were calculated using the ΔΔCt method. The assay can detect a minimum of 10 copies of spiked FIV plasmid per reaction, equivalent of a theoretical minimum of 350 copies per ml of supernatant.

### 2.4 Standard PCR

The presence of FIV viral DNA in infected cells was confirmed by using PCR and the primers LTR forward 3 (5′-GCTTAACCGCAAAACCACAT-3′) and GAG reverse 3 (5′-CAAATCTCCTGGCTTGAAGG-3′) amplifying a 466 base pair region between the 5′ LTR and *gag* of the FIV genome. Primers that bind to feline glyceraldehyde 3-phosphate dehydrogenase (GAPDH) gene were used as control for equal DNA loading (GAPDH forward–5′-CCTTCATTGACCTCAACTACAT-3′; GAPDH reverse–5′-CCAAAG TTGTCATGGATGACC-3′). All reactions used GoTaq^®^ Flexi DNA Polymerase kit (Promega, USA) as per manufacturer's protocol with the following cycling parameters: An initial denaturation step of 3 min at 95 °C was followed by 35 cycles of denaturation at 95 °C for 45 s; annealing at 57 °C for 45 s and extension at 72 °C for 1 min. The amplification was completed with a final extension step at 72 °C for 10 min. PCR products were visualised by 2% agarose gel electrophoresis followed by ethidium bromide staining.

### 2.5 Stimulation of FIV production from IL-2 depleted MYA-1 T-cells by Prostratin and other chemicals

MYA-1 T-cells were seeded at a density of 5 × 10^5^ cells/ml in the absence of IL-2 and 24 h post-seeding the cells were infected with FIV GL8 at multiplicity of infection (M.O.I.) of 0.01 unless stated otherwise. They were then washed twice and resuspended in fresh RPMI without IL-2 supplement. The IL-2 depleted cells were stimulated with either PMA (0.08), Prostratin (1 μM), and/or Gö6850 (2.5 μM) or mock stimulated with solvent μM (DMSO) 2 days post-infection (unless otherwise stated). The stimulatory effects of the phorbol esters were titrated and the concentrations of 0.08 μM PMA and 1 μM Prostratin were selected as optimal. The concentration of 2.5 μM of Gö6850 was used based on (Martiny-Baron et al., 1993) and is a concentration that has been reported to block both conventional and novel isoforms of PKC. RGV and AZT were used at 1 μM and 5 μM respectively where indicated. Samples were collected at the indicated time points and the removed volume was replaced with a matched volume of medium containing the appropriate compounds. Samples were stored at −80 °C until the end of the experiment at which time virus levels in the culture supernatants were measured by either an enzyme-linked immunosorbent assay (ELISA) for FIV capsid antigen (CA, p24) (IDEXX PetCheck anti FIV antigen, IDEXX Laboratories, ME, USA), or by a non-isotopic reverse transcriptase activity assay (Lenti RT kit, Cavidi AB, Sweden) as per manufacturer's instructions. Absorbance was measured spectophotometrically using a Multiskan Ascent Plate reader (Labsystems).

### 2.6 FIV productive infection assay

IL-2-supplemented MYA-1 T-cells were seeded at a density of 5 × 10^5^ cells/ml and infected with FIV GL8 (M.O.I. = 0.01) for 2 h at 37 °C. Cells were then washed and resuspended in medium supplemented with IL-2 and stimulated with Prostratin (1 μM) and/or Gö6850 (2.5 μM) at 2 days post infection. Supernatant samples were taken at day 8 post infection to be assayed for reverse transcriptase activity or FIV p24 concentrations. Cell growth and viability was measured by Trypan Blue exclusion assay at day 8 post infection.

### 2.7 Flow cytometry

Flow cytometry was used to measure expression of FIV receptors CD134 and CXCR4 on MYA-1 T-cells. 0.5 μg of primary antibodies (the mouse anti-feline CD134 monoclonal antibody 7D6 (Willett et al., 2007) and the Mouse anti-feline CXCR4 monoclonal antibody (R&D Systems, Minneapolis, USA)) were used to label their respective receptors. Secondary staining of the receptors was performed using 0.5 μg of Rabbit anti-mouse IgG RPE secondary antibody (AbD Serotec, Oxford, UK). Processed cells were then analysed by flow cytometry using an EPICS MCS-XL flow cytometer (Beckman Coulter, High Wycombe, UK), with 10,000 events being collected for each sample in LIST mode. Data was analysed using EXPO 32 ADC Analysis software (Beckman Coulter).

### 2.8 Statistics

Data was analysed for statistical significance using the one way analysis of variance (ANOVA) test with the Bonferroni and the Tukey *post-hoc* means comparison tests from OriginPro 8.6 (OriginLab Corp., USA) where indicated.

## 3 Results

### 3.1 Effect of phorbol esters on IL-2-depleted T-cells infected with FIV

**Fig. 1:**
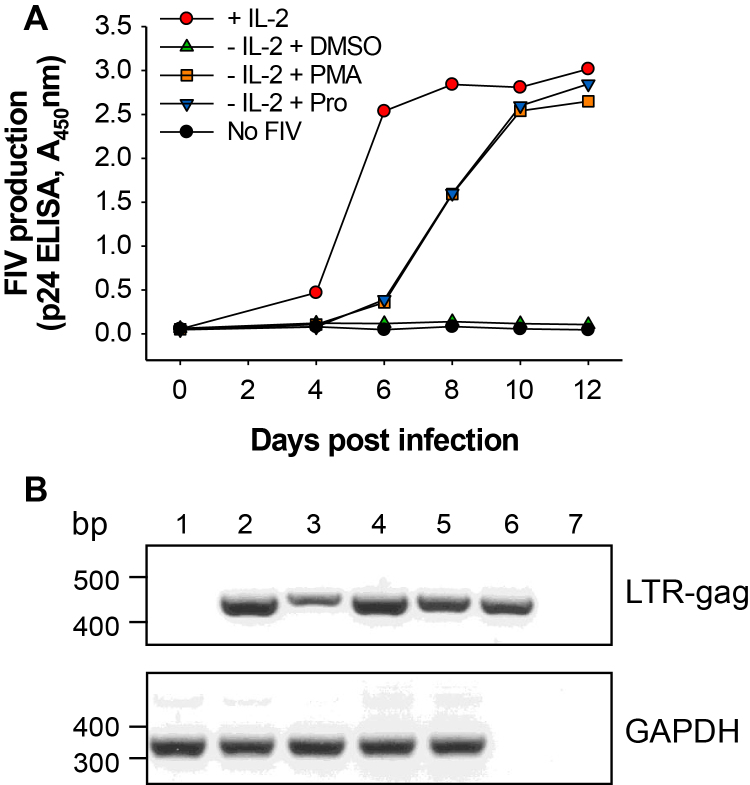
Prostratin stimulates productive FIV infection from infected, IL-2-depleted T-cells. (A) IL-2 depleted, FIV GL8-infected (M.O.I. = 0.01) MYA-1 CD4^+^ T-cells were stimulated with either PMA, Prostratin (Pro) or mock stimulated (DMSO) 4 days after infection and virus production was measured by FIV p24 ELISA. (B) DNA was extracted from FIV infected cells at day 12 post infection and screened by PCR for either a 466 bp FIV LTR–gag product or a GAPDH control. Lanes: (1) −FIV, +IL2; (2) +FIV, +IL-2; (3) +FIV, −IL-2, +DMSO; (4) +FIV, −IL-2, +PMA; (5) +FIV −IL-2, +Prostratin, (6) FIV plasmid positive control, (7) dH_2_O negative control.

In the presence of IL-2, FIV replicated in the MYA-1 T-cells, with the FIV capsid protein (p24) level in the supernatant reaching a plateau six days post infection (Fig. 1A). When cells were depleted of IL-2 24 h prior to infection, FIV production was dramatically reduced (Fig. 1). The addition of Prostratin or the tumour-promoting phorbol ester, phorbol myristate acetate (PMA), after infection to the IL-2-depleted, FIV-infected cells stimulated virus production (Fig. 1A). Standard PCR was used to confirm the presence of FIV DNA within the total extracted cellular DNA of the IL-2-depleted, mock-treated T-cells at the end of the experiment, although it should be noted that this PCR did not confirm the integration of the provirus (Fig. 1B).

**Fig. 2:**
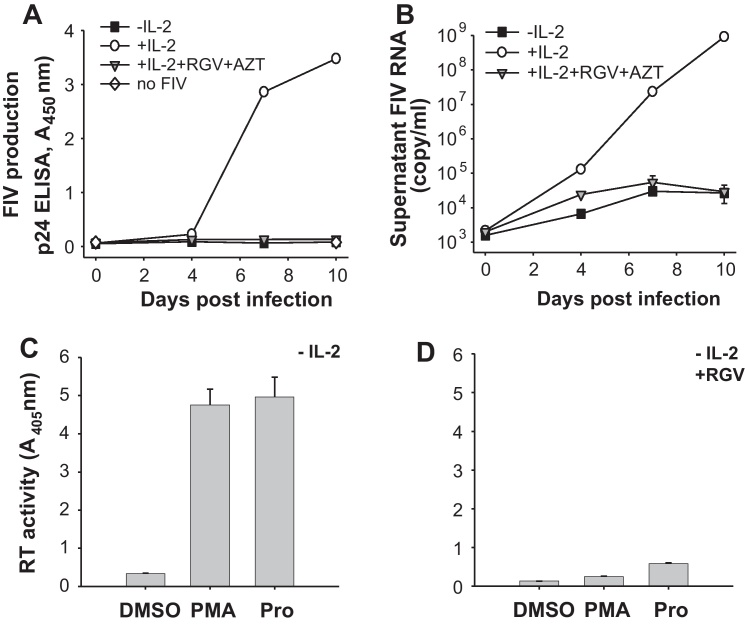
*De novo* infection contributes to most of the virus production detected by our assay. MYA-1 T-cells supplemented with or depleted of IL-2 for 24 h were infected with FIV GL8 (M.O.I. = 0.01). Virus production was measured using (A) FIV p24 ELISA and (B) qPCR. The antiretrovirals RGV and AZT were added to appropriate samples 24 h after infection. Each point represents mean ± standard error for the p24 ELISA data and mean ± standard deviation (*n* = 3) of the calculated copy number for the qPCR data and representative of two independent experiments. (C) IL-2-depleted MYA-1 CD4^+^ T-cells were infected with FIV GL8 (M.O.I. = 0.06). Two days after infection the cells were stimulated with PMA, Prostratin (Pro) or mock stimulated (DMSO). Supernatant was collected at day 10 post-infection and virus production was quantified by a reverse transcriptase (RT) activity assay. (D) Parallel experiment to (C) in which RGV was added to the FIV-infected cells 24 h post-infection and 24 h prior to treatment with PMA or Prostratin. Each bar represents the mean ± standard error (*n* = 3) and representative of three independent experiments.

To assess the contribution of *de novo* infection with FIV to the overall virus production as measured by our assay, a parallel infection assay was performed in the presence of the retroviral integrase inhibitor Raltegravir (RGV) and the reverse transcriptase inhibitor Zidovudine (AZT) at concentrations previously shown to be effective against FIV (Bisset et al., 2002; Savarino et al., 2007). The antiretroviral drugs were added 24 h post infection, thus allowing virus production from cells infected during the initial inoculation but blocking any subsequent reverse transcription and integration. The application of the antiretroviral drugs rendered FIV replication from IL-2-supplemented cells undetectable by p24 ELISA (Fig. 2A). Only by using the more sensitive technique of qPCR a residual level of virus production was detected in both IL-2-depleted and antiretroviral-treated, IL-2 supplemented cells (Fig. 2B). In a similar experiment, when IL-2 depleted, infected cells were simultaneously treated with RGV and PMA or Prostratin, virus replication was dramatically reduced compared with cells not treated with RGV (Fig. 2C and D). These results indicate that in our assay the majority of the virus production driven by IL-2 or by the phorbol esters was the result of *de novo* infection of adjacent cells.

### 3.2 Effect of phorbol esters on MYA-1 CD4^+^ T-cell growth and viability

**Fig. 3:**
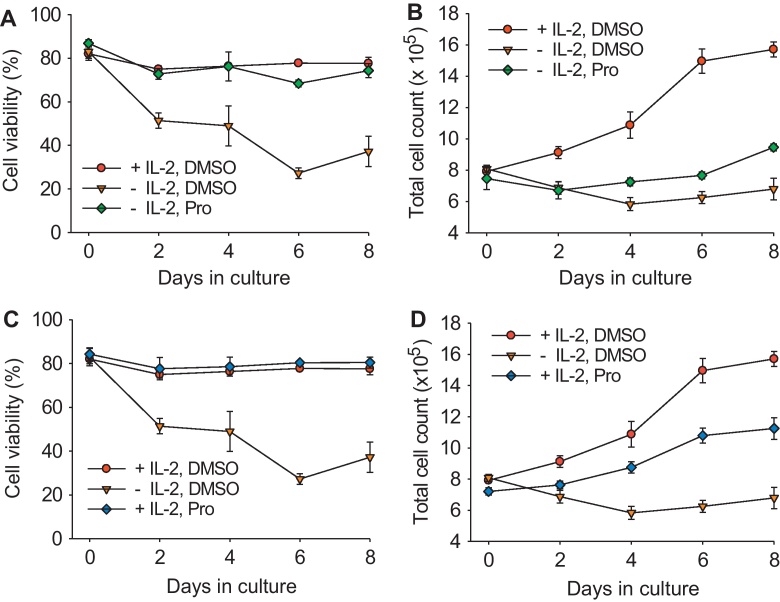
Effect of Prostratin on cell growth and viability. (A) Cell viability and (B) total cell count of IL-2-depleted MYA-1 CD4^+^ T-cells stimulated with Prostratin (Pro) or mock stimulated (DMSO) compared with IL-2-supplemented cells treated with DMSO. (C) Cell viability and (D) total cell count of IL-2-supplemented cells stimulated Prostratin or mock stimulated compared with IL-2-depleted cells treated with DMSO. Each point represents the mean ± standard error (*n* = 3) and representative of three independent experiments. Cell viability was measured by Trypan Blue exclusion.

In the absence of exogenous IL-2, cell growth ceased and viability fell to between 20 and 30% within six days (Fig. 3A and C). In contrast, the addition of exogenous IL-2 maintained the viability of the cells at approximately 80% and resulted in a doubling of cell number over the eight-day period (Fig. 3B and D). The addition of Prostratin prevented the drop in the viability of IL-2-depleted cells during the study period (Fig. 3A) and triggered a modest increase in cell number (Fig. 3B). Furthermore, the addition of Prostratin in the presence of IL-2 had little effect on cell viability but reduced cell growth, with the treated cultures achieving a final density of 1.1 × 10^6^ cells/ml compared with 1.6 × 10^6^ cells/ml in the untreated cultures (Fig. 3D). These data concur with previous observations describing the cytoprotective properties of Prostratin in human cells (Gustafson et al., 1992; Korin et al., 2002).

### 3.3 Reactivation of virus production from IL-2-depleted cells by Prostratin appears to require protein kinase C (PKC)-dependent signalling

**Fig. 4:**
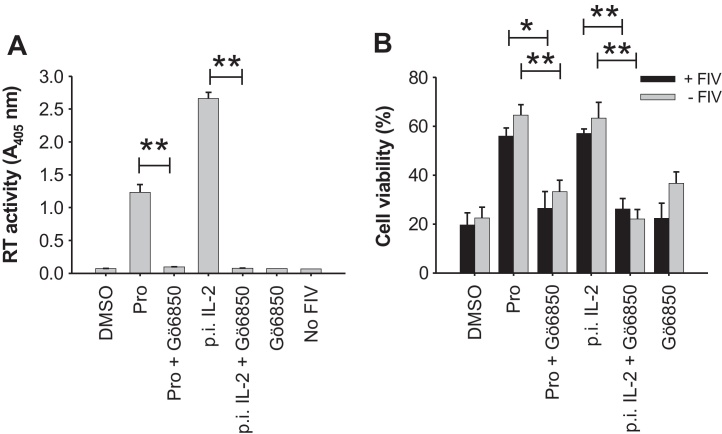
IL-2 or Prostratin-driven FIV replication requires PKC. IL-2-depleted MYA-1 CD4^+^ T-cells were infected with FIV GL8 (M.O.I. = 0.01). Two days post-infection, cells were stimulated with Prostratin (Pro), Prostratin plus Gö6850 (Pro + Gö6850), IL-2 (100 U/ml), IL-2 + Gö6850, Gö6850 or mocked stimulated (DMSO). Control uninfected cells were maintained in the absence of IL-2 (−FIV) and (A) virus production was monitored by a reverse transcriptase (RT) activity assay. (B) Viability of the cells receiving each treatment (as in (A)) in the presence or absence of FIV was measured at 8 days post-infection by Trypan Blue exclusion. Each bar represents the mean ± standard error (*n* = 3) and results are representative of three independent experiments. (p.i.) indicates IL-2 was added post infection (*p < 0.05; **p < 0.01).

Prostratin is a potent activator of the PKC signalling pathway in human cells and its ability to reactivate latent HIV-1 has been attributed to stimulation of this pathway (Williams et al., 2004). We therefore asked whether the specific inhibitor of PKC “Gö6850”, a compound that targets both conventional and novel PKC isoforms, would inhibit the stimulatory effects of Prostratin *in vitro*. The application of Gö6850 (at a concentration of 2.5 μM, which should inhibit most novel and conventional isoforms of PKC (Martiny-Baron et al., 1993)) significantly reduced the stimulation of FIV production by Prostratin in IL-2-depleted MYA-1 cells (Fig. 4A). The ability of exogenous IL-2 to reactivate virus production from IL-2-depleted cells was similarly abrogated by Gö6850 (Fig. 4A), indicating a common mechanism of action of Prostratin and IL-2 in the stimulation of FIV replication. Treatment with Gö6850 also significantly reversed the cytoprotective effect of Prostratin and IL-2, suggesting a correlation between the maintenance of cellular viability and the induction of viral replication (Fig. 4B) and this was consistent with the vital modulatory role of PKC in T-cell activation and IL-2-dependent signalling (Isakov and Altman, 2002; Tan and Parker, 2003).

### 3.4 In the presence of IL-2, Prostratin inhibits productive infection with FIV

**Fig. 5:**
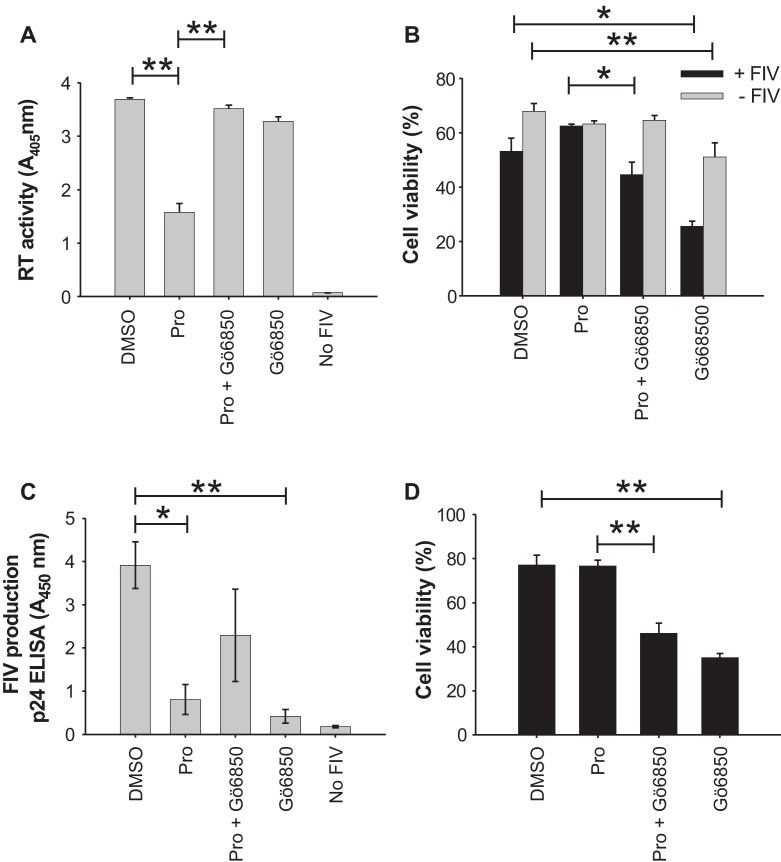
Inhibition of productive infection with FIV by Prostratin in the presence of IL-2 is PKC-dependent. MYA-1 CD4^+^ T-cells were cultured in the presence of IL-2 and infected with FIV GL8 (M.O.I. = 0.01). Prostratin (Pro), Prostratin and Gö6850 (Pro + Gö6850), Gö6850, or solvent (DMSO) were added to the cells 2 days post-infection. At day 8 post-infection, (A) virus production was quantified in the culture supernatant by a reverse transcriptase (RT) activity assay while (B) the viability of the infected and un-infected cells was assessed by Trypan Blue exclusion. Each bar represents the mean ± standard error (*n* = 3) and is representative of two independent experiments. In separate experiments, virus production was quantified at day 8 post-infection by FIV p24 ELISA (C) and the viability of FIV infected cells were assessed by Trypan Blue exclusion (D). Each bar represents the mean ± standard error (*n* = 4) and the data are the combination of two experiments (*p < 0.05; **p < 0.01).

In addition to its role in reversing HIV-1 latency, Prostratin had been shown to inhibit productive infection with HIV-1 in human cells (Biancotto et al., 2004; Kulkosky et al., 2001; Rullas et al., 2004; Warrilow et al., 2006). We therefore assessed whether this phenomenon extended to FIV infection of feline cells. In the presence of IL-2, Prostratin significantly reduced FIV production from infected MYA-1 CD4^+^ T-cells in all experiments (Fig. 5A and C). Furthermore, in two independent experiments the restriction of virus production was reversed completely by the simultaneous treatment of the cells with Gö6850, while data from two other independent experiments also showed Gö6850 mediating a reversal of viral restriction but it was not statistically significant (Fig. 5C). These observations suggest that the inhibitory effect required PKC-dependent signalling. An assessment of cell viability (Fig. 5B and D) indicated that Prostratin did not reduce the number of viable cells and thus the inhibitory effect on virus growth was not the result of a non-specific toxic effect. Importantly, the addition of Gö6850 alone did not increase productive infection relative to mock stimulated cells (Fig. 5A and C), instead virus replication was significantly reduced in 2 out of 4 experiments (Fig. 5C) and the viability of infected cells was significantly reduced in all experiments.

**Fig. 6:**
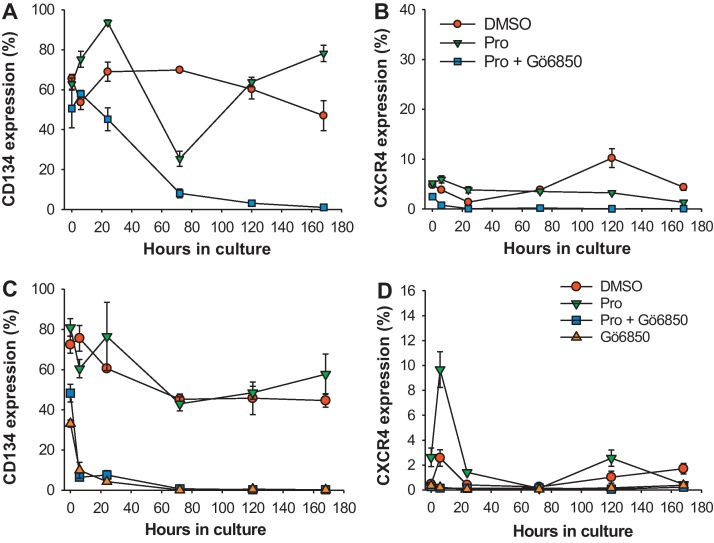
Effect of Prostratin on the expression of CD134 and CXCR4 on CD4^+^ T-cells. MYA-1 CD4^+^ T-cells were cultured in IL-2-supplemented medium in the presence of Prostratin (Pro), Prostratin plus Gö6850 (Pro + Gö6850), or solvent (DMSO). Sequential samples were collected over a 7 day period and the expression of (A) CD134 and (B) CXCR4 was quantified by flow cytometry. Data is representative of three independent experiments. In an additional experiment the effect of applying Gö6850 alone on the expression of CD134 (C) and CXCR4 (D) was investigated. Each point represents the mean ± standard error (*n* = 3).

Previous studies have suggested that the inhibition of productive infection with HIV-1 by Prostratin was mediated by the down-regulation of HIV-1 receptors CD4, CCR5 and CXCR4 (Biancotto et al., 2004; Kulkosky et al., 2001; Rullas et al., 2004; Warrilow et al., 2006). We therefore examined the effect of Prostratin on the expression of the FIV receptors CD134 (Shimojima et al., 2004) and CXCR4 (Willett et al., 1997) on MYA-1 T-cells in the presence of IL-2 over a 7 day period. The expression of CD134 showed a general decline over the experimental period from ∼70% to ∼40% positive (Fig. 6A and C), while the expression of CXCR4 on the CD4^+^ T-cells was relatively low compared with CD134 (Fig. 6B and D). Prostratin had variable effects on the expression of CD134 and CXCR4 between experiments, which was probably a reflection of the nature of the MYA-1 CD4^+^ T-cells as they cycle through phases of blasting, rapid expansion and then rest. However, when Prostratin and Gö6850 were added to the cells, a treatment which rescued FIV replication from Prostratin-induced inhibition, the expression of both CD134 and CXCR4 was dramatically reduced to below the detection limit of flow cytometry (Fig. 6). The application of Gö6850 alone to the cells also rapidly reduced the expression of both CD134 and CXCR4 (Fig. 6C and D). Thus the ability of Prostratin to inhibit productive infection with FIV cannot be explained by the down-regulation of expression of either CD134 or CXCR4.

**Fig. 7:**
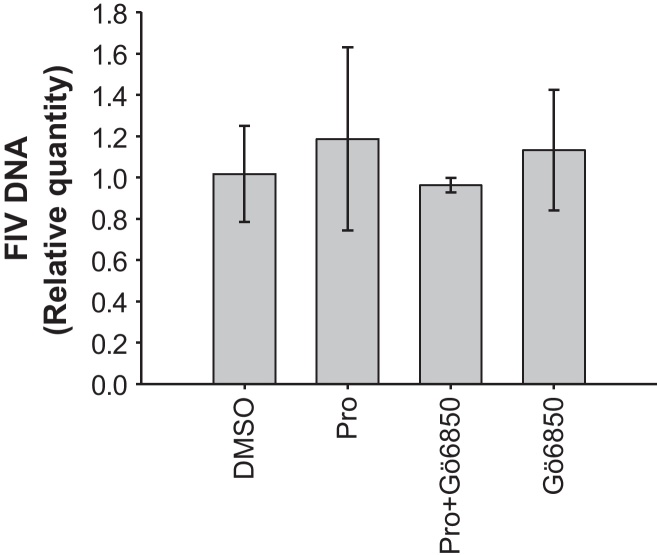
No reduction in intracellular FIV DNA following pre-treatment of CD4^+^ T-cells with Prostratin. IL-2 supplemented MYA-1 CD4^+^ T-cells were pre-incubated with Prostratin (Pro), Prostratin plus Gö6850, Gö6850 alone or mocked treated (DMSO) for 24 h before infection with FIV GL8. 2 h post infection the cells were washed twice and total cellular DNA extracted. qPCR amplifying a region of the FIV *gag* gene was performed on the DNA samples and the results were analysed using the ΔΔCt method. The data were relative to the average ΔCt value of the mock treated (DMSO) cells. Each bar represents the mean ± standard deviation of the calculated relative quantities (*n* = 3) and is representative of three independent experiments.

Finally, to assess the effect of Prostratin and Gö6850 on viral entry directly, IL-2-supplemented MYA-1 CD4^+^ T-cells were pre-incubated with Prostratin, Prostratin plus Gö6850, Gö6850 or DMSO for 24 h prior to infection with FIV. Total cellular DNA was extracted 2 h post infection and intracellular FIV DNA was quantified by qPCR. Results from three independent experiments indicated that similar amounts of FIV DNA were found in cells subjected to each of the four treatments (Fig. 7), which further supports the conclusion that Prostratin is acting post-entry to inhibit FIV replication.

## 4 Discussion

Despite advances in the development of antiretroviral drugs, HIV-1 remains a major concern for global public health. Research into the molecular biology of HIV-1 and related retroviruses such as FIV (which can be used as a comparative model for HIV) may lead to novel means to control or even cure the disease. The curious dual effect of Prostratin on the growth of HIV-1 has been studied by various laboratories and Prostratin or other PKC modulating compounds could be used potentially to stimulate latent HIV-1 reservoirs within the body (Richman et al., 2009). In this study, we investigated the effect of Prostratin on the replication of FIV *in vitro* and we found many parallels, as well as some differences, between the interaction of FIV and HIV-1 with their respective host cells.

Viruses often encode accessory genes, the products of which are used to manipulate the host cell in order to enhance viral replication; for example, the HIV-1 Vpr protein mediates cell cycle arrest in order to increase the transcription of the provirus (Romani and Engelbrecht, 2009). However, virus replication is also vulnerable to global changes in cell signalling, as demonstrated here by the effect of withdrawing IL-2 on FIV replication. The reliance of FIV on the cytokine and signalling of the host cell is analogous to the replication dynamics of HIV-1, which also requires a T-cell activation signal, including those driven by IL-2 (Oswald-Richter et al., 2004; Stevenson et al., 1990; Williams et al., 2004). The IL-2-dependency of FIV infection in MYA-1 T-cells mirrors the behaviour of CD4^+^CD25^+^ T-cells isolated from feline peripheral blood mononuclear cells, which could be non-productively infected by FIV in the absence of IL-2 (Joshi et al., 2005). The similarities between the two systems suggest that the MYA-1 CD4^+^ T-cell culture system described herein reflects the characteristics of CD4^+^ T-cells *ex vivo*. As we are constantly striving to reduce the requirement for the use of animals in biomedical research, MYA-1 T-cells may alleviate the need for primary feline CD4^+^ T cells to perform initial validation studies of novel compounds for use in the reversal of viral latency.

By administering the antiretroviral drugs RGV and AZT post-infection, we established that most of the virus produced in our assays came from the ability of Prostratin or IL-2 to promote *de novo* infection after an initial burst of virus production. The withdrawal of IL-2 not only inhibited *de novo* FIV infection, it also stopped cell growth and caused a drop in cell viability. In the absence of IL-2, Prostratin appears to be mimicking the function of IL-2 by rescuing virus spread and cell viability.

When MYA-1 T-cells were supplemented with IL-2, productive FIV infection was inhibited by Prostratin. This viral restriction, plus the aforementioned stimulatory effect of Prostratin are both reversed by the PKC inhibitor Gö6850, indirectly suggesting that both of these processes are mediated by PKC. This mirrors similar findings in HIV-1 studies (Williams et al., 2004). PKCs are vital to T-cell receptor signalling and T-cell activation (Isakov and Altman, 2002). Because both HIV-1 and FIV target activated CD4^+^ T-cells, the viruses have evolved to exploit the cellular pathway of T-cell activation by encoding binding sites for several transcription factors of the PKC signalling cascade in their genomes: the HIV-1 LTR contains sites for NFAT, NFκB, and AP-1, while the FIV LTR contains sites for AP-1, AP-4 and ATF (Sparger et al., 1992; Tang et al., 1999; Thompson et al., 1994). In addition, we also observed that Gö6850 abrogated the enhancement of FIV spread and the cyto-protective effect by exogenous IL-2, suggesting that IL-2 and Prostratin may modulate cell growth and FIV spread by signalling through the same PKC-dependent pathway. However, Gö6850 could not reliably inhibit productive infection driven by IL-2. Furthermore, Gö6850 reversed the Prostratin mediated virus restriction in all bar one experimental replicate. We believe that there are two likely explanations for this variability of results: firstly, unlike truly transformed cell lines with relatively uniform growth dynamics, MYA-1 cells retain some of the characteristics of primary cells as they undergo phases of blasting, expansion and rest. This variability may translate to variable cellular PKC activation levels in between experiments, which give rise to the variability of the effect of Gö6850 on virus replication. Secondly, there is considerable redundancy within the IL-2 signalling network. IL-2 may signal down pathways other than PKC such as the JAK/STAT5, MAPK and PI3K pathways (Cheng et al., 2011). We have found no studies which address directly the effect of PKC blockage on IL-2 stimulated HIV-1 production and further experimental work is required to confirm the involvement of the aforementioned pathways in IL-2-dependent lentivirus production. Future research should also attempt to directly demonstrate the role of PKC in this phenomenon and to identify the isoform(s) responsible.

Based on these findings we propose that there is an optimal level of PKC activation for FIV spread and replication. We speculate that in a state of low PKC activation such as after the removal of exogenous IL-2, Prostratin becomes a substitute for IL-2 and raises PKC activation back to the optimum level. In contrast, if the cells are already supplemented with IL-2, the addition of Prostratin would over-stimulate PKC, to the detriment of FIV spread and replication. What then is the mechanism behind the PKC-mediated viral restriction? There was no reduction in viability after the IL-2-supplemented cells were treated with Prostratin, thus it is unlikely that Prostratin inhibits virus replication by a non-specific toxic effect. Prostratin had a variable effect on the expression of CD134 and CXCR4, which we attribute to the non-uniform nature of the MYA-1 cell growth. The simultaneous addition of Gö6850 and Prostratin to cells led to the rapid down regulation of both viral receptors, yet the application of Gö6850 rescued productive infection from Prostratin-induced inhibition. Thus down-regulation of the FIV receptors cannot be invoked as a reason to explain the FIV-inhibitory effect of Prostratin.

The down-regulation of CD4, CCR5 and CXCR4 has been observed consistently among Prostratin-stimulated human CD4^+^ T-cells, supporting the hypothesis that Prostratin's anti-HIV-1 effect is mediated at the viral entry step (Biancotto et al., 2004; Gulakowski et al., 1997; Kulkosky et al., 2001; Rullas et al., 2004). Does this suggest that Prostratin blocks HIV-1 and FIV *via* distinct mechanisms? While the entry of viral pseudotypes bearing HIV-1 Env (but not VSV-G protein) is inhibited by Prostratin (Rullas et al., 2004), blockade of the FIV receptors demonstrated by a pseudotype assay does not necessarily translate to inhibition of productive infection (Willett et al., 2009). It is possible that Prostratin induces changes to the viral receptor expression levels among activated CD4^+^ T-cells that can disrupt pseudotype entry, but the magnitude of change is too small to block infection with replication-competent virus. Further, the data presented herein demonstrated that 2 h after infection, Prostratin-stimulated cells contained similar amounts of intracellular viral DNA compared with mock-treated cells, suggesting that viral entry and reverse transcription were not affected by Prostratin. This contrasts with two studies of Prostratin's effect on HIV-1 infection of human CD4^+^ T-cells, which found that reverse transcription and HIV-1 p24 uptake was inhibited (Biancotto et al., 2004; Warrilow et al., 2006). These findings may indicate that Prostratin has distinct effects on HIV-1 and FIV infection. Alternatively, differences in the experimental protocol may account for these discrepancies, for example, in one of the studies Prostratin was washed off before HIV-1 infection (Warrilow et al., 2006), whereas in all our experiments the drugs remained in the supernatant throughout the experiment. Prolonged exposure to phorbol ester is known to down-regulate PKC (Liu and Heckman, 1998). However, the down regulation of PKC by phorbol esters is a highly variable phenomenon, with different isoforms of PKC been differentially affected within different cell types (Liu and Heckman, 1998). Currently we do not know which PKC isoform is down regulated, for how long and by how much in MYA-1 cells. Also we do not know whether any potential PKC down regulation can inhibit virus replication. Furthermore, we observed no evidence of down regulation affecting virus replication when FIV-infected IL-2-depleted cells were treated with PMA and Prostratin. Moreover, down regulation could not explain the rescue of virus replication using the PKC-inhibitor Gö6850, which is presumably the functional equivalent of a complete down regulation of PKC. It is possible that there is an as yet uncharacterised PKC-driven antiviral mechanism operating in both human and feline cells. The existence of an entry-independent inhibitory mechanism mediated by phorbol esters was proposed previously for HIV-1 when it was found that phorbol esters could inhibit virus replication in PBMCs at concentrations that did not reduce HIV-1 receptor expression significantly (Warrilow et al., 2006). Our discovery of an entry-independent inhibition of FIV by Prostratin in feline cells supports the existence of such a mechanism.

To our knowledge this is the first demonstration of Prostratin's antiviral activity against a virus other than HIV-1. While the identity of the underlying mechanism in feline cells remains elusive at present, further research on whether Prostratin's effect on the cell and lentivirus replication is warranted, especially if Prostratin or other PKC-modulatory compounds are to be developed as an anti-HIV latency treatment.

## Competing interests

The authors declare that they have no competing interests.

## Author's contributions

CNC performed the experiments with the occasional assistance of ELM. CNC and BJW designed the study, while BJW and MJH supervised all aspects of the research project. All authors reviewed the final data and manuscript.
